# Makerere University College of Health Sciences’ role in addressing challenges in health service provision at Mulago National Referral Hospital

**DOI:** 10.1186/1472-698X-11-S1-S7

**Published:** 2011-03-09

**Authors:** Irene B Kizza, Joshua Tugumisirize, Raymond Tweheyo, Speciosa Mbabali, Arabat Kasangaki, Edith Nshimye, Juliet Sekandi, Sara Groves, Caitlin E Kennedy

**Affiliations:** 1 School of Health Sciences, College of Health Sciences, Makerere University, Kampala, Uganda; 2 School of Medicine, College of Health Sciences, Makerere University, Kampala, Uganda; 3 School of Public Health, College of Health Sciences, Makerere University, Kampala, Uganda; 4 Department of Community/Public Health, Johns Hopkins School of Nursing, Baltimore, 21205, USA; 5 Department of International Health, Johns Hopkins Bloomberg School of Public Health, Baltimore, 21205, USA

## Abstract

**Background:**

Mulago National Referral Hospital (MNRH), Uganda’s primary tertiary and teaching hospital, and Makerere University College of Health Sciences (MakCHS) have a close collaborative relationship. MakCHS students complete clinical rotations at MNRH, and MakCHS faculty partner with Mulago staff in clinical care and research. In 2009, as part of a strategic planning process, MakCHS undertook a qualitative study to examine care and service provision at MNRH, identify challenges, gaps, and solutions, and explore how MakCHS could contribute to improving care and service delivery at MNRH.

**Methods:**

Key informant interviews (n=23) and focus group discussions (n=7) were conducted with nurses, doctors, administrators, clinical officers and other key stakeholders. Interviews and focus groups were tape recorded and transcribed verbatim, and findings were analyzed through collaborative thematic analysis.

**Results:**

Challenges to care and service delivery at MNRH included resource constraints (staff, space, equipment, and supplies), staff inadequacies (knowledge, motivation, and professionalism), overcrowding, a poorly functioning referral system, limited quality assurance, and a cumbersome procurement system. There were also insufficiencies in the teaching of professionalism and communication skills to students, and patient care challenges that included lack of access to specialized services, risk of infections, and inappropriate medications.

Suggestions for how MakCHS could contribute to addressing these challenges included strengthening referral systems and peripheral health center capacity, and establishing quality assurance mechanisms. The College could also strengthen the teaching of professionalism, communication and leadership skills to students, and monitor student training and develop courses that contribute to continuous professional development. Additionally, the College could provide in-service education for providers on professionalism, communication skills, strategies that promote evidence-based practice and managerial leadership skills.

**Conclusions:**

Although there are numerous barriers to delivery of quality health services at MNRH, many barriers could be addressed by strengthening the relationship between the Hospital and MakCHS. Strategic partnerships and creative use of existing resources, both human and financial, could improve the quality of care and service delivery at MNRH. Improving services and providing more skills training could better prepare MakCHS graduates for leadership roles in other health care facilities, ultimately improving health outcomes throughout Uganda.

## Background

Uganda, like many of its sub-Saharan African neighbors, faces multiple and competing health challenges that put significant stress on the country’s health system [1]. To make progress on core health indicators and respond to the health needs of the country in the future, Uganda’s government, institutions, and partners must emphasize health systems strengthening and strategic planning. With one trained health worker per 1,818 people, no clear guidelines to coordinate and optimize the use of existing health workers, and no clear leadership in how best to train additional health workers, Uganda has serious human resource needs [2]. Furthermore, health facilities and equipment are in a state of disrepair, existing infrastructure is insufficient for core functions and most health education curricula emphasize curative medicine to the detriment of preventive medicine [2].

The new Health Sector Strategic Plan III (HSSP III) states that achieving the nation’s health goals based on current needs will require effective health care delivery, relevant and adequate research capacity, effective health programs, and relevant and informed health policies. The HSSP III (2010-2015) builds on the achievements of the prior plans and was developed through an intensive collaborative process across multiple stakeholders. A key component of HSSP III is universal delivery of the Uganda National Minimum Health Care Package, which focuses on prevention, management and control of communicable diseases (malaria, STI/HIV/AIDS), health promotion, disease prevention and community health initiative including epidemics and disaster preparedness and response, and maternal child health and nutrition.

Makerere University College of Health Sciences (MakCHS) trains a variety of health sciences students, including nurses, physicians, pharmacists, radiologists and public health practitioners. MakCHS is affiliated with a variety of national and regional referral hospitals and health centers, including Mulago National Referral Hospital (MNRH). Mulago Hospital, one of the largest hospitals in East Africa with 1,500 bed capacity, is the main training hospital in Uganda. Its mission is to improve the health of the people of Uganda and beyond and promote health equity by providing quality education, research and health services. MakCHS and MNRH have a close relationship. MNRH is the central teaching hospital for MakCHS students, and many MakCHS faculty members are engaged in direct service delivery at MNRH [3]. MNRH is a tertiary care facility, but due to patient demands, it currently functions as both a primary health care facility and a general specialty hospital, and only to a limited extent as a tertiary care facility.

MakCHS participates in care and service delivery at MNRH in a variety of ways. College staff members provide direct clinical care and supervise students in their clinical rotations at the hospital and through institutions that are directly affiliated with the University and MNRH, such as the Infectious Diseases Institute (IDI). College staff works in MNRH laboratories. Post-graduate students contribute through clinical placements at MNRH, during which students improve standards of care on the units by asking questions of their supervisors and conducting operational research to improve daily service delivery practice. MakCHS further contributes to service delivery through partnerships with government departments, namely the Ministry of Health (MoH) and local government health centers, by providing expert opinions on government health guidelines and policies. Care and service provision also occurs through College staff-initiated projects, which may be oriented towards research, service, or education. The presence of students in clinical practice promotes modeling of professionalism and evidence-based practice. MakCHS has a role in research, teaching, and care and service delivery through use of evidence-based practice [4].

In 2009, a partnership was created between Makerere University and Johns Hopkins University (MU-JHU) to build and sustain MakCHS as a transformative institution and a leader in Africa [1]. To contribute to health systems strengthening and strategic planning, a task force was appointed as part of a needs assessment to examine the current state of care and services in selected HSSP priority areas at MNRH. Specifically, the goal of this study was to identify the challenges and gaps in care and service provision in selected HSSP priority areas at MNRH, and identify opportunities for MakCHS to contribute to addressing these challenges and gaps.

## Methods

This study was conducted in Kampala, Uganda at MNRH and MakCHS. A qualitative approach was selected to allow for an open-ended exploration of the issues. Data were collected through key informant interviews (KIIs) and focus group discussions (FGDs) with health care providers and administrators at MNRH and MakCHS. While students and patients are also important stakeholders, this study focused on providers and administrators who tend to be more familiar with the structure of the relationship between MNRH and MakCHS and the accompanying institutional challenges and opportunities for improvement.

Respondents were purposively sampled to cover the care and service units responsible for HSSP priority areas: HIV/AIDS, malaria, tuberculosis, maternal health, child health, and mental health. Within these units, respondents were further selected to represent different levels of providers (heads of departments, doctors, nurses in-charge, nurses, midwives, and a pharmacist). In total, 23 individual interviews and 7 focus groups were conducted. Details about participant characteristics are presented in Table 1.

**Table 1:** Participant characteristics

HSSP priority area	Method	Number conducted	Job categories of participants	Total participants
Maternal Health	FGD	3	• Midwives	20
	KII	5	• Clinical Head MOH • Head MU • Unit in Charges	5

Child Health	KII	3	• Clinical Head MOH • Head MU • Unit in Charges	3

Mental Health	KII	3	• Clinical Head MOH • Head MU • Unit in Charges	3

HIV	FGD	2	• IDI Staff	11
	KII	1	• Clinical Head IDI	1

Malaria	FGD	1	• Clinical Officers	6
	KII	3	• Unit in Charge • Doctor in charge, Assessment Center • In-charge Lab Assessment Center	3

Tuberculosis	FGD	1	• Nurses	6
	KII	2	• Doctor in Charge • Unit in Charges	2

Cross-topic	KII	7	• Head DOM • Clinical Head DOM • SPNO • Principal pharmacist • Principal MakCHS • Records in charge • Deputy Director MNRH	7

DOM = Department of Medicine

SPNO = Senior Principal Nurse Officer

For both interviews and focus groups discussions, semi-structured guides were developed and used to lead discussions. These guides included questions about the range of care and services offered in the unit; gaps in care and services offered; quality of care perspectives and quality improvement measures; key outputs; challenges in care and service delivery; and opportunities for improvement. Interviews and focus group discussions were conducted in English by IK, JT, RT, AK, EN, and SM in private locations on the Hospital and College campus. Interviews typically lasted thirty minutes to one hour, and focus groups for 60 to 90 minutes.

Interviews and focus groups were audio-taped with the permission of the respondents and transcribed verbatim, usually by the original interviewer. Transcripts were read in their entirety, and themes were developed through an iterative, collaborative process with the study team, including all of the original interviewers. Summary statements with representative quotes were developed for each theme. Matrices were created to examine emergence of identified themes across participant types and HSSP areas. The final analysis was shared and discussed among all co-authors, who approved the findings.

All respondents were informed of the purpose, risks, and benefits of participation, and written informed consent was obtained prior to participation. Institutional review boards at both the Johns Hopkins Bloomberg School of Public Health and the Makerere University School of Public Health approved this study.

## Results

Respondents identified a number of challenges and gaps in care and service provision at MNRH. However, they also suggested potential solutions and areas where MakCHS could contribute to improvements in care and service delivery at MNRH. We describe these challenges and proposed solutions below.

### Quality assurance and patient safety

#### Challenges

##### Quality of services

Most respondents during interviews and focus groups said that quality of services in MNRH is sub-optimal, but given the limited resources, the Hospital is doing its best.

“Effectiveness of our services here in Mulago . . . Of course we have a long way to go . . . we are doing our best but in a situation where you are having underfunding and inadequate supplies, inadequate drugs, inadequate diagnostics . . . I think we need better funding and improved facilities to meet the needs of the population around. Everyone can see that.” (KII, provider)

With a few exceptions, most providers were unfamiliar with the quality assurance measures used currently in their units. In this study quality assurance referred to a system for monitoring the quality of care, identifying problems, and instituting changes to resolve those problems for continuous improvement.

##### Overcrowding

One major challenge to quality assurance and patient safety was overcrowding. Respondents said overcrowding reduces the time available for quality client-patient interaction across all priority areas and prevents adequate monitoring of seriously ill patients. Outpatient clients frequently wait for long hours to receive care, and many leave the hospital without ever receiving care. Overcrowding is also a source of stress and burnout among staff.

##### Infection control

Another challenge affecting patients’ and providers’ safety is infection control. The concept of patient safety here focused on limiting adverse events that caused the patients harm. Some respondents said that infection control measures in the hospital are inadequate. On the medical wards, there are many immune-compromised patients, yet few measures are enacted to protect patients and providers from tuberculosis (TB). “We do not know how much we are spreading TB,” said one administrator, while other administrators and providers acknowledged that there is no service for treatment of multi-drug resistant tuberculosis.

Providers in one focus group discussion agreed that there are too few autoclaves and that existing autoclaves occasionally break down, leading to unsatisfactory sterilization of equipment. Another administrator said there are limited hand washing facilities that work. A few providers noted that heavy workloads affect adherence to strict infection control measures and treatment guidelines. One administrator mentioned that there are insufficient programs available to vaccinate providers, and staff lack knowledge about personal protective equipment.

#### Solutions

Existing quality assurance methods described by respondents in some departments included regular audits, management meetings, case conferences, mortality review meetings, case reviews, seminars and workshops, and academic discussions, while other units relied solely on informal feedback from patients. Respondents from units with a stronger research focus said that quality of care and services provided was high. All providers from the HIV/AIDS unit mentioned that there are formal mechanisms in place to ensure quality, such as a monitoring and evaluation unit, a functional quality control team, a quality care improvement team with specific indicators and a tracking system, and stipulated intervals for conducting client satisfaction surveys. These strategies could be shared across units and implemented where they are lacking. One administer suggested that existing systems for quality assurance could be strengthened, and another suggested that MakCHS should interact with MNRH to improve quality at all levels.

### Health providers

#### Challenges

##### Knowledge and skills

Respondents reported inadequate programs and facilities to equip health providers with knowledge and skills regarding new developments in the practice of medicine. Providers have limited opportunities for refresher training and exposure to best practices outside Uganda. As one described:

“Me with the other old, old information, I am still carrying it in my head. So we need updates . . . Maybe if we could access internet and read things ourselves, then we can talk knowing that I read it on internet and in such and such a country, so and so did a research and came up with this.” (FGD, provider)

##### Balancing care and teaching

Respondents felt that senior staff members were frequently absent from their duties and failed to adequately supervise MakCHS students on clinical rotations and offer supportive supervision to junior providers. Respondents in child health, medicine, and maternal health said that with the large patient care load, staff found it challenging to balance clinical care with teaching responsibilities.

##### Motivation and performance

Many respondents mentioned low motivation among providers due to excessive workload, lack of teamwork, lack of timely promotions, low salary, and limited incentives, such as housing, meals and transport allowances. One administrator pointed out that research projects tend to attract both newly trained and older doctors from the clinical practice in health care facilities because they pay more competitively. Lack of encouragement and support from both the hospital and the community further reduced morale.

“I feel the good points about the health worker should be supported and encouraged by the clients and employers. And when that is done, you find that someone is being motivated more. But there is something that goes on in our communities with the clients, that in case something slight happens, you find that people want to go and talk about it on radio and saying like health workers are doing bad and I see this is discouraging.”

(FGD, provider)

The inadequate working environment also reduced staff motivation.

“If I am coming to work I should be given a white coat, a blood pressure machine, a stethoscope, a blood glucose testing machine, there should be a wall clock which I can use to take patients respiration rates and pulses . . . those are the issues . . . simple things . . . stationery (laughter).” (KII, provider)

Lack of consultation and involvement of providers in planning for a variety of areas – including procurement of correct and appropriate supplies, modification of infrastructure and general managerial issues – was also mentioned by providers in one focus group as reasons for lack of motivation.

##### Lack of professionalism

Due to the many challenges faced by staff, several respondents felt that there was limited professionalism among staff. In this study professionalism specifically meant following an ethical standard, showing respect, good interpersonal communication, the performance of assigned duties as scheduled, collaboration with the health team, and good time management. One provider focus group agreed that senior staff fail to adequately supervise medical students during clinical rotations, and are frequently absent or late in reporting for duty. Two administrators mentioned that communication with patients is inadequate and partly due to heavy workload, especially among nurses and midwives. Incidences of under-the-counter fees to health providers were mentioned during one provider focus group, and participants in a different group cited lack of team spirit as an additional challenge.

“Much as we are saying that we are a team there is a way medical officers tend to discriminate the clinical officers, clinical officers also tend to discriminate the others. There is that bit of marginalization” (FGD, providers)

Some respondents felt that the lack of professionalism and quality of care made them not want to use the Hospital themselves.

“It’s embarrassing that many of us, when we have relatives, family members sick, we just don’t go to Mulago and yet that’s where we work; that’s where the highest expertise is, but we then opt to take our family members and relatives outside to other hospitals and private clinics. That is embarrassing; they are voting with their feet and saying I don’t want what I am seeing or what I am participating in.” (KII, administrator)

##### Stress management

A few respondents identified the need for stress management programs for staff in view of the heavy workload and stressful nature of clinical positions.

“As counselors, we absorb a lot of information from so many people . . . The stress needs to be dealt with . . . We have not reached burnout, but there are no stress management programs in place.” (FGD, providers)

#### Solutions

To improve staff knowledge levels, respondents suggested opening a library for staff and financing staff refresher trainings. Several respondents noted that this was an area where MakCHS could contribute by leading training and refresher sessions for providers at MNRH including sessions on professionalism, leadership, and quality assurance.

Respondents also recommended a variety of incentives to improve providers’ morale and performance. The single most important issue identified was the need for the institution to provide adequate drugs, equipment, supplies and personal protective equipment in order to make the workplace a safe environment. Some focus group participants proposed transport reimbursement, providing meals, and salary increases as well. Respondents also suggested that the College could develop stress management programs for providers at MNRH.

To improve professionalism among staff, some respondents felt that senior faculty and providers should endeavor to mentor and nurture professional conduct among junior staff. They also suggested that the ethics committee should be empowered to improve its functionality. The College could contribute by helping develop MNRH ethics committees for research and clinical services and provide role models. Additionally, one interviewee noted that the ethics course is taught early within the medical curriculum and should instead be taught throughout training.

### Resource constraints

#### Challenges

Without exception, all administrators and providers across all priority areas mentioned that available resources were inadequate to provide the level of care and service required.

##### Human resource shortages

MNRH has a severe provider shortage across all HSSP priority areas. This shortage is particularly acute among nurses and laboratory technicians, and it impacts the quality of care and services provided.

“The nurses are few. At night, nurse to patient ratio is one per 40 or 50 patients while during day it is one nurse per 25 or 30 patients. What kind of nursing service do you expect?” (KII, administrator)

##### Supplies and equipment shortages

Many respondents, including administrators and providers across HSSP priority areas, mentioned an inadequate supply of equipment such as gloves, cannulae, intravenous sets, stationery, laboratory diagnostics, drugs, blood and intravenous fluids, beds, blood pressure machines, and fetoscopes. The inadequate and intermittent supply of drugs was said to result in resistant diseases, especially multi-drug resistant tuberculosis, and relapses of mental disorders. Health education materials, such as booklets, pamphlets, and television to play videos, are also lacking.

Respondents in some units said that resources to ensure patient comfort, such as bed linen, blankets, and laundry services, are inadequate. Many respondents also mentioned that patient nutrition is also inadequate, and that this affected patient outcomes.

“You can’t expect a TB patient to improve so fast and you leave out the diet . . . diet is so, so part and parcel of the treatment . . . how will the bones gain weight? How will they improve?” (KII, provider)

One administrator pointed out that the repair and maintenance of high quality equipment such as CT scans and X-ray machines are a challenge for which the staff is not prepared.

##### Transport shortage

Respondents in mental health, tuberculosis, and maternal health units said that community outreach services were deficient, primarily because of inadequate transport. Many respondents who had done community outreach work had used public transportation, leading to wasted time and limited coverage of the catchment area.

#### Solutions

Participants noted vacant positions both at MakCHS and MNRH should be filled. However, additional government funding through either the Ministry of Education and Sports or the MoH was still necessary for staff recruitment. Participants in both interviews and focus groups also said that the MoH and MakCHS should provide competitive pay packages in order to attract and retain high-quality health providers and educators. One administrator suggested that joint appointments be created between MNRH and MakCHS to help in addressing this shortage. To ensure that qualified providers have adequate competencies including laboratory skills, respondents suggested that the College curriculum be revised to allot more time to clinical and laboratory teaching, and design strategies to hold senior staff accountable for student teaching, supervision and assessment.

Although severe resource shortages are a reality at MNRH, respondents felt that in some areas, improvements could be made through more efficient use of existing space and resources. For example, one respondent in pediatrics reported they almost exclusively receive 500 mls intravenous fluid packs, yet they need 250 mls pediatric packs. This means they discard 250 mls each time, a waste of resources that could be avoided if individual units were consulted for the appropriate procurement. During a focus group, providers mentioned that many drugs for less common health problems are procured but expire before use.

Several respondents argued that MakCHS should explore opportunities to develop the capacity of the public primary care facilities within Kampala city, making the referral system more functional. The majority of interview respondents said that if the referral system was improved through collaboration with other providers, overcrowding and congestion would be solved.

Participants also suggested sharing best practices across units. Benchmarking and setting a standard for best practices should be hospital policy. MNRH could draw from experiences of good practices from IDI that were developed in collaboration with MakCHS. Similarly, participants recommended pilot-testing creative programs to improve quality, then expanding these programs across the hospital if they prove successful. For example, one participant suggested that a patient nutrition program should be piloted in one of the units with greatest need, and then expanded if the program proved successful. Other participants suggested strengthening outreach services, but this would require additional funding and staff

To increase finances and resources overall, participants suggested that MNRH should consider partnering with development agencies such as the UN World Food Program and industry corporate social responsibility programs. Also they suggested that MNRH could write grants to increase resources. MakCHS could help spearhead this through training in grant writing and assisting with applications.

### Specialization

#### Challenges

##### Insufficient specialized services

Although MNRH is meant to serve as the tertiary medical facility for Uganda, many respondents said it currently serves more of a primary care role because many patients seek care for basic health needs. This has resulted in the overloading of MNRH, wasting resources designated for tertiary care on primary care concerns. In addition, the hospital is unable to provide many specialized services because it lacks the necessary supplies and equipment or specially trained personnel. For example, in the medical department, providers mentioned lack of specialized diagnostics and therapeutics such as laser therapy, therapeutic endoscopy and MRI scanning. In mental health, there are few psychiatrists specializing in pediatrics and obstetrics, and in child health, there are few specialists in pediatric oncology, gastroenterology, renal, cardiology and pulmonary. One administrator said that without these specialized services in MNRH, it is impossible for MakCHS to train specialists for the country.

#### Solutions

The majority of respondents felt that the referral system should be re-established and the capacity of peripheral health units should be strengthened. This would allow Mulago to spend more time and resources on its role as a tertiary health facility. MakCHS could contribute as a training institution by providing more specialized training opportunities for different cadres of staff, including nurses. These opportunities may be obtained through partnering with collaborators. However, such changes should be made considering all training needs. One administrator said that as MNRH focuses on becoming more specialized, it will lose its ability to train undergraduate students in general and primary care. It was suggested that the College may need to look for additional sites for this type of training.

### Relationship between MNRH and MakCHS

At the time of this study, there was no clear memorandum of understanding between MNRH and MakCHS. Clearly defined roles for each party were not specified, which caused challenges in the working relationship between the two institutions. As one administrator stated:

“We are still working on a gentleman’s agreement . . . It is not easy to work in such a situation. If we proceed in the way we are working today, it is going to be very dangerous.” (KII, administrator)

The lack of a clearly defined working relationship seemed to have the greatest impact on resources. Many respondents, both providers and administrators across different units, mentioned that MNRH bears the majority of costs related to student learning, and several suggested that MakCHS should supplement Mulago Hospital for materials used.

## Discussion

Many of our study findings are consistent with the challenges and gaps in service delivery identified in the HSSP III [2] and Uganda Service Provision Assessment Survey [5]. Issues of inadequate or nonexistent quality assurance mechanisms, inefficient use of available resources, poor provider motivation, lack of adherence to professional codes of conduct, deficient management and leadership skills, inadequacies of human resource in terms of numbers and skills mix, inadequate support supervision and continous professional development, low remuneration, inadequate infection control measures, weak referral system, inadequancy of medicines and supplies and inadequate health financing are some of the findings that have been similarly reported from other settings. This study adds to this literature by helping to identify how MakCHS can play a role in addressing challenges across these areas.

The first major challenge faced by MNRH was quality assurance. In developing countries, the risk for health associated infections is twenty times higher than in developed countries because of poor social environment, deficient infrastructure, rudimentary equipment, and a lack of national and local infection control policies [6]. A strong surveillance system would allow MNRH to collect data about the burden of health care-associated infection and could guide identification of locally effective interventions necessary for the safety of patients and health workers.

Within MNRH, there was limited experience with quality improvement procedures. The MoH developed a quality assurance program in 1994, and support supervision guidelines were published in 2000. Among our respondents, however, there was limited familiarity with quality assurance methods.

The University, however, has established a quality assurance directorate. At MakCHS, a committee has been charged with developing *Standards for Health Professional Training Institutions*. Draft copies have been reviewed and a final set of standards will be published soon. The College could be an excellent resource in operationalizing a quality assurance program at the hospital.

The Infectious Disease Institute (IDI) at Makerere University is an accredited international centre for management of HIV/AIDS, and has a fully functioning quality assurance program that can serve as a model. Some of the main elements of their program include having a quality manager, a functioning quality assurance committee, an information management system, performance standards and outcome measures, measurement tools, regular audits, continuous quality monitoring, feedback from the clients, international accreditation audits, and specific financial resources for quality assurance. The IDI is committed to the use of treatment guidelines and protocols. Safety procedures are enforced, and critical incident analysis practiced. Other units at MNRH could learn from the quality assurance program in place at the IDI.

Previous experience suggests that for a quality assurance program to succeed after implementation, it must have specific ongoing resources committed to support an information management system, a team dedicated to quality assurance, and a means to distribute information [7]. The Yellow Star program in Uganda and another quality assurance program in Zambia were set up with donor funds and were well received, but without continued funding, both programs proved unsustainable [7].

Second, staff burnout is a challenge for MNRH. The lack of commitment from senior MakCHS and MoH staff demonstrated by absenteeism, neglecting duties, illness, low productivity, and non-attendance at teaching sessions may be related to occupational stress. Previous studies have suggested that the main sources of stress among providers are understaffing, dealing with too many very sick patients, crowded wards, lack of resources, lack of control, difficult work schedules, work overload, poorly defined roles, inability to create change, inadequate security, a culture of blame, and poor career advancement and salaries [8,9]. Findings from this study suggest that the MNRH environment is characterized by all the stressors cited by Thomas and Valli [8]. The inappropriate functioning of MNRH as a primary health care facility was also mentioned as a source of stress, especially for senior consultants. No studies have measured levels of occupational stress at MNRH, and neither MNRH nor MakCHS have staff stress management programs or job performance assessment mechanisms.

To manage stress and reduce risk of staff burnout, MakCHS could offer in-service training on stress and its effects, coping strategies (e.g. time management, task prioritization, delegation), creating a support system, developing a greater sense of control, and relaxation [8]. Involving staff in interventions and decision making has been shown to improve clinical outcomes with little cost. MakCHS can also assist with some changes in the organizational structure: committed providers, a functional occupational health and safety committee, clear job descriptions, continuing professional education and skills development opportunities, management training for departmental heads, and regular performance assessment with career planning. Finally, screening providers for burnout using a validated tool should be considered [10].

A third challenge for MNRH is professionalism. Ample evidence exists that professional character is formed in health professions schools and shaped by influential factors including formal teaching and informal processes like medical rounds, peer interactions, and role models. MakCHS and MNRH must show commitment towards teaching professionalism by ensuring a well-structured, integrated curriculum with well-defined learning activities and evaluated outcome competencies. Teaching should be both theory-based in a classroom and experiential in the clinical area. Professionalism must be actively taught and promoted with the objective of transforming students from members of the lay public to expert members of a profession with both appropriate skills and commitment to a set of values. Deans, heads of departments and program directors need to manifest their support through allocation of space, teaching time and financial resources as well as recognition of those who participate. At MakCHS, committed and trained staff are needed to facilitate teaching and learning and demonstrate desired roles [11]. MakCHS and MNRH must ensure through faculty development that teachers have the necessary knowledge and skills to both teach and role model professionalism [12], and their efforts must be recognized and rewarded by the institutions [11]. Professionalism must be taught throughout the curriculum at both the undergraduate and postgraduate levels [13-15]. The cognitive base and experiential learning appropriate for each stage of learning should be included in all major teaching units to ensure continuity [11]. Additionally, MakCHS and MNRH should develop a mechanism for evaluating student learning.

Role modeling is at the heart of transmitting professional values and attitudes. The unavailability and behavior of senior staff may affect role modeling and mentoring of professionalism. Repeated negative student experiences have an adverse impact on the learner’s development of professionalism [16]. This consequently affects the quality of patient care. At MNRH, several challenges interfere with being a good mentor: large patient loads, competing demands of teaching and providing service, and the difficulty in developing and evaluating valid strategies for transmitting professionalism [16]. In this study, respondents said that provision of care for many patients with few clinical staff reduced time available for teaching good communication skills, role modeling positive patient-provider interactions and demonstrating humanistic patient care.

At MakCHS, training in professional conduct for all students is done only in the first year of study. The course includes communication, ethics, and patient-provider interaction. Such training should be integrated throughout the entire curriculum. In addition, there is a need to identify how the curricular professional competencies are taught and evaluated for both MakCHS undergraduates and residents.

A fourth issue that arose in this study was leadership and management of human resources. Poor leadership and management of human resources constitute major barriers to quality health care delivery at all health system levels [17]. Issues of lack of training opportunities for staff, lack of a reward system, lack of mechanisms to ensure sense of accountability and responsibility among staff and poor staff motivation may be related to management and leadership and may contribute to high rates of absenteeism and dualism among staff resulting in low productivity. There is a need to strengthen the management and leadership skills of the managers and leaders at MakCHS and MNRH.

Finally, financial constraints pose a challenge to MNRH. Participants in this study identified financial constraints as a major issue contributing to the quality of care and services provided in the hospital and to limitations in training of health professionals. According to Uganda’s National Health Policy [17], the MoH has several functions: resource mobilization and budgeting, setting standards for quality assurance, capacity development, and technical support.

Participants in this study said that a minority of the essential medicines and health supplies required and requested for the basic health care package were budgeted by the central government. The shortage in allocation of funds to the health sector affects all levels of health care facilities, negatively affecting the quality of health care [7]. A study about priority setting in health care institutions conducted in 2006 in an Ugandan hospital showed that of a US$ 32.4 million estimated hospital budget for the financial year 2006/2007 only about 30% was received [18]. Sufficient funding is a pivotal factor in infrastructure, drug supply, and staff recruitment and retention. Political commitment through adequate funding for health resources is necessary to ensure quality training of health workers and health care delivery for Ugandans.

In a situation where the demand for health services outstrips the available resources, priority setting has been identified as a useful tool for resource allocation [18]. A 2006 study described financial priority setting in a teaching hospital in Uganda using an ethical framework, *Accountability for Reasonableness*, to assess and evaluate the priority setting processes [18]. The authors observed a lack of knowledge about the process and concluded that priority setting decisions at the hospital did not satisfy the conditions of fairness. They recommended that the hospital: (1) engage frontline practitioners, (2) publicize the reasons for decisions both within the hospital and to the general public, and (3) develop formal mechanisms for challenging the reasoning. In addition, capacity strengthening was required for senior managers to accept responsibility for ensuring that the above three conditions were met. It may be relevant to conduct a similar study for both MakCHS and MNRH to evaluate how priority-setting decisions are made and how those decisions influence both health care delivery and teaching.

Findings from this study should be examined in the context of its limitations. This study was limited to a single institution, MNRH, which serves as a unique tertiary and teaching hospital within Uganda. Findings may not be transferrable to other institutions in Africa or elsewhere. Also, due to limited time, we were only able to conduct single interviews and focus groups with a limited number of respondents. However, this study also had notable strengths. By conducting a qualitative research study as part of a strategic planning process, we had access to key informants who were knowledgeable, open, and willing to discuss the issues. Adequate time was allotted for interviews and focus group discussions. Analysis was conducted by the original interviewers with guidance and expert input from qualitative specialists, and collaboration in the analysis process strengthened the final interpretation of results through researcher triangulation. Saturation of the data was achieved, as respondents repeatedly referred to the same challenges and potential solutions across respondents working in different selected HSSP priority areas, with different levels of training, and who participated in different data collection methods. Finally, this qualitative study was complemented by other data collection methods, including a patient exit survey and examination of clinic records. This triangulation across data collection techniques helped strengthen our confidence in the results.

## Conclusions

There are numerous challenges to care and service delivery at MNRH, all of which are linked in some way to the limited available resources. The capacity of MNRH is overstretched to breaking point. MakCHS has a huge challenge of providing quality education, research, care and services in an environment of limited resources. In spite of this, MakCHS has and can continue to make significant contributions to the capacity of MNRH to address HSSP priorities. MakCHS should strengthen its partnership with MNRH so that both institutions collaborate in planning and development. Improving services, addressing quality assurance and providing more skills training could better prepare MakCHS graduates for leadership roles in other health care facilities, ultimately improving health outcomes throughout Uganda.

## List of abbreviations used

FGD: focus group discussion; HSSP: Health Sector Strategic Plan; IDI: Infectious Disease Institute; KII: key informant interview; MakCHS: Makerere University College of Health Sciences; MNRH: Mulago National Referral Hospital; MoH: Ministry of Health; MU: Medical Unit; MU-JHU: Makerere University and Johns Hopkins University; DOM: Department of Medicine; SPNO: Senior Principal Nurse Officer.

## Competing interests

The authors declare that they have no competing interests.

## Authors’ contributions

All of the authors of this study were funded by a grant from the Bill and Melinda Gates Foundation. IK participated in the conception and design of the study, participated in the data analysis, and led the drafting of the manuscript. JT participated in the conception and design of the study and data analysis and helped draft part of the manuscript. RT participated in the data analysis and helped draft part of the manuscript. AK participated in the design of the study and data analysis. SM and EN participated in the conception, design and data analysis. JS participated in the conception and design. SG and CK participated in data analysis and the draft of the manuscript. All authors read and approved the final manuscript.
